# Modeling human liver organ development and diseases with pluripotent stem cell-derived organoids

**DOI:** 10.3389/fcell.2023.1133534

**Published:** 2023-02-15

**Authors:** Rie Ouchi, Hiroyuki Koike

**Affiliations:** ^1^ Institute of Research, Tokyo Medical and Dental University (TMDU), Tokyo, Japan; ^2^ Department of Biochemistry and Molecular Biology, Nippon Medical School, Tokyo, Japan

**Keywords:** liver, organoids, pluripotent stem cells, disease modeling, regenerative medicine, developmental biology, drug screening

## Abstract

The discoveries of human pluripotent stem cells (PSCs) including embryonic stem cells and induced pluripotent stem cells (iPSCs) has led to dramatic advances in our understanding of basic human developmental and cell biology and has also been applied to research aimed at drug discovery and development of disease treatments. Research using human PSCs has been largely dominated by studies using two-dimensional cultures. In the past decade, however, *ex vivo* tissue “organoids,” which have a complex and functional three-dimensional structure similar to human organs, have been created from PSCs and are now being used in various fields. Organoids created from PSCs are composed of multiple cell types and are valuable models with which it is better to reproduce the complex structures of living organs and study organogenesis through niche reproduction and pathological modeling through cell-cell interactions. Organoids derived from iPSCs, which inherit the genetic background of the donor, are helpful for disease modeling, elucidation of pathophysiology, and drug screening. Moreover, it is anticipated that iPSC-derived organoids will contribute significantly to regenerative medicine by providing treatment alternatives to organ transplantation with which the risk of immune rejection is low. This review summarizes how PSC-derived organoids are used in developmental biology, disease modeling, drug discovery, and regenerative medicine. Highlighted is the liver, an organ that play crucial roles in metabolic regulation and is composed of diverse cell types.

## 1 Introduction

**FIGURE 1:**
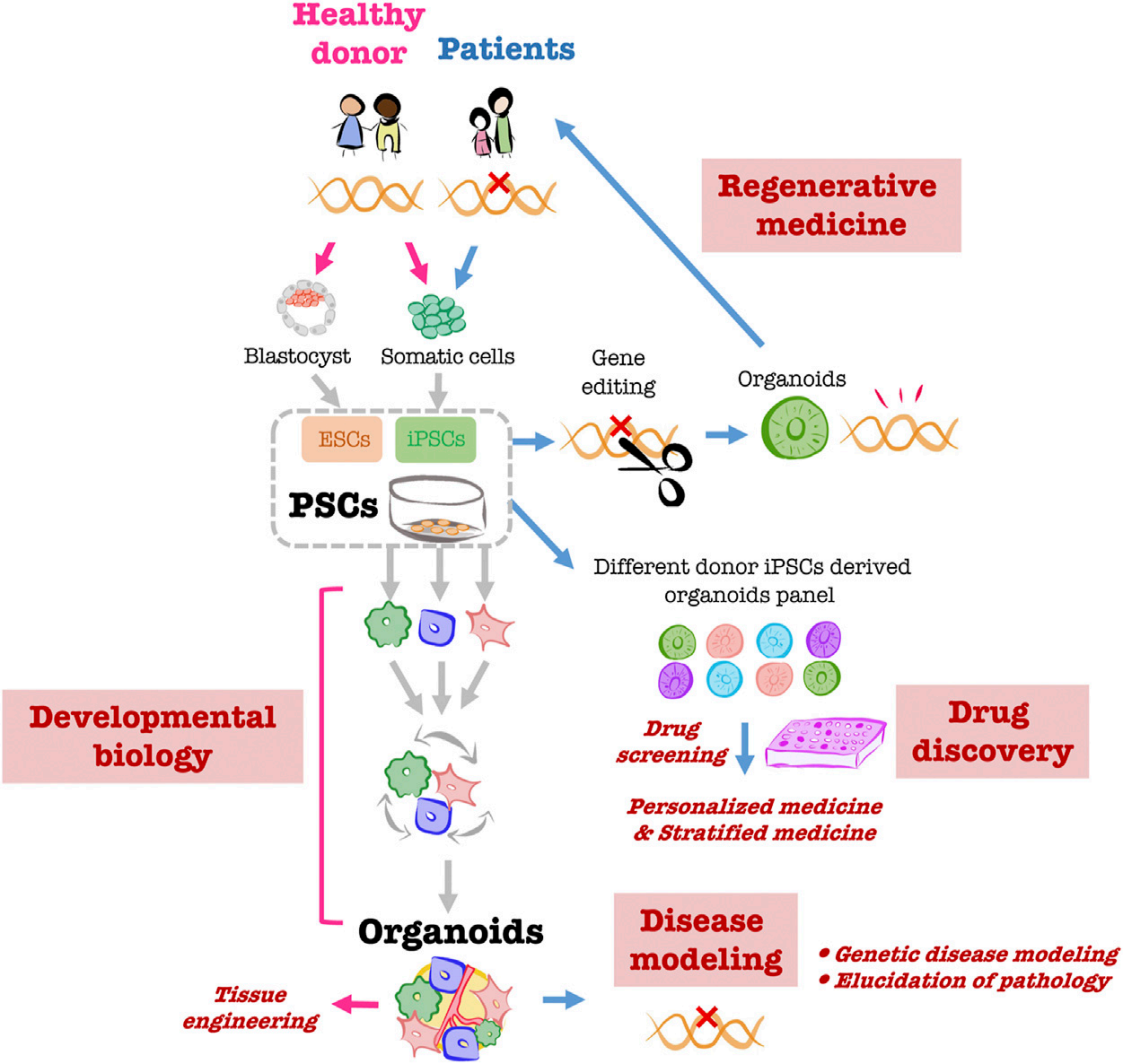
Overview of PSC-derived liver organoid applications Creating organoids using PSCs obtained from healthy donors enables the *in vitro* reproduction of human organogenesis through which multiple cells coexist and build up during development. The organoids created through this process can be used as helpful *ex vivo* models for tissue engineering. Organoids created from patient-derived iPSCs will be utilized for pathological modeling. It is anticipated that organoid panels created from multiple human iPSCs will be used as platforms for drug screening and other applications and will be applied to personalized and stratified medicine. Moreover, gene editing of patient-derived iPSCs will enable the creation of organoids for transplantation into the same patient.

Organoids are *ex vivo* “organ-like” tissues exhibiting three-dimensional structures that closely resemble the structures and functions of corresponding organs *in vivo*. Human organoids have been created that reproduce the intestinal tract, brain, kidneys, lungs, and liver (Kim et al., 2020). The major cell sources for human organoids include primary tissue stem cells isolated from biopsy specimens as well as human pluripotent stem cells (PSCs), including human embryonic stem cells (ESCs) isolated from human blastocysts (Thomson et al., 1998) and human induced pluripotent stem cells (iPSCs) established by introducing four transcription factors into human fibroblasts (Takahashi et al., 2007). Organoids created from human PSCs have the following advantages (Figure 1). 1) Whereas primary cells from *in vivo* organs are difficult to obtain, PSCs can be used to generate any number of required human cell types. 2) Because PSCs can generate different cell types, organoids made from them are able to internalize multiple cell types, including mesenchymal, hematopoietic, and epithelial cells, making the complexity of the constituent cells close to that of organs *in vivo*. Such complexity recapitulates the intricacies of organs and diseases, as well as the cell-cell interactions and niches significant for forming organs (Armingol et al., 2021). This makes PSC-derived organoids a valuable model with which to study human development, especially organogenesis. 3) iPSCs can be obtained minimally invasively through harvest from peripheral blood (El Hokayem et al., 2016), and they inherit the genetic background of the donor. Consequently, the creation of organoids from human iPSCs reflects the characteristics of each donor and can be applied to personalized and stratified medicine. Moreover, the creation of organoids from patient-derived iPSCs can be used for disease models, disease elucidation, and drug screening that reflects the genetic background underlying the pathological condition. 4) Genome editing of iPSCs can be used to replace gene mutations that cause diseases as well as for disease modeling and clarification of pathological conditions. In addition, organoids derived from iPSCs with human leukocyte antigens (HLAs) that are less susceptible to immune cell attack could potentially be used as transplant replacement organs with less risk of immune rejection (Morizane et al., 2017).

The liver is the largest organ in the body and performs a variety of essential functions, including the metabolism of sugars, lipids and proteins; production of bile; and regulation of blood homeostasis, including glucose levels (Reinke and Asher, 2019). Because the liver is formed through the coordinated development of diverse cell types, it is anticipated that, among other advances, the creation of liver organoids from human PSCs through mixing of multiple cell types will enhance our understanding of the essential processes contributing to organogenesis in the human liver and to the identification of cells and factors essential for organogenesis. In addition to parenchymal cells, including hepatocytes and bile duct cells, the liver is composed of multiple non-parenchymal cells, including vascular cells and Kupffer cells (liver-resident macrophages) as well as mesenchymal cells, which are all known to play various roles in the progression of chronic liver diseases such as non-alcoholic steatohepatitis (NASH) and alcoholic fatty liver disease (AFLD). This makes human PSC-derived liver organoids composed of multiple cell types a valuable model with which to recapitulate those pathological conditions. In addition, because the liver plays a central role in drug metabolism, human PSC-derived liver organoids have the potential for use in evaluating drug safety and efficacy on a large scale, which has been difficult with experimental animal models and primary human stem cells due to differences in responsiveness among animal species and the difficulty in obtaining such cells. Moreover, given the shortage of donors for liver transplantation (Kwong et al., 2021), iPSC-derived liver organoids, with their high viability and low risk of immune rejection, may represent a promising option for regenerative medicine. In this review, therefore, we will address the current progress in research on organoids derived from PSCs, especially iPSCs, dividing the discussion into areas of developmental biology, disease modeling, drug discovery and regenerative medicine. A spotlight will be placed on the liver, an organ for which there is significant potential demand for human PSC-derived organoids.

## 2 Main text

### 2.1 Developmental biology

Developmental studies carried out mainly in mice have identified FGF, BMP, retinoic acid, and WNT signaling as essential factors in early hepatic induction (Zorn, 2008). Studies using human PSCs have also shown that these molecules mediate essential signaling pathways inducing the differentiation of human endoderm cells into hepatocyte-like cells, and two-dimensional culture systems are reasonable models for studying differentiation into hepatic endoderm cells (Touboul et al., 2010). However, two-dimensional culture system are not able to mimic and explain the mechanisms underlying development from the viewpoint of liver organogenesis, in which immature hepatocytes detach from the foregut endodermal sheet and infiltrate the septum transversum mesenchyme (STM) to form a condensed tissue mass, which eventually becomes vascularized and matures through interaction with surrounding cells (Zaret, 2002; Han et al., 2020). It was therefore necessary to establish a three-dimensional system in which cells were adjacent to one another.

In 2013, Takebe et al. induced hepatic endoderm from human iPSCs and found that when human umbilical vein endothelial cells (HUVECs) and mesenchymal stem cells were co-cultured on Matrigel beds, the cells autonomously constructed three-dimensional human liver organoids (Takebe et al., 2013; Takebe et al., 2014). This self-organization underlying liver organoid formation was achieved through direct cell-to-cell interactions (Asai et al., 2017). Subsequently, this group also successfully generated all of the component cells comprising the organoid from iPSCs (Takebe et al., 2017a; Sekine et al., 2020). Interestingly, organoids with iPSC-derived STM and vascular endothelial cells (ECs) had more advanced liver characteristics than organoids developed from HUVECs and mesenchymal stem cells. Several co-culture experiments have shown the usefulness of PSC-derived STM and PSC-derived ECs, especially liver sinusoidal ECs, for maturation of PSC-derived hepatocyte-like cells (Koui et al., 2017; Ardalani et al., 2019; Miyoshi et al., 2019; Raggi et al., 2022). This enhanced maturation of hepatocytes may have been achieved because liver organoids with a mixture of STM and ECs recapitulates the organogenesis niche (Sosa-Pineda et al., 2000; Margagliotti et al., 2007) in which liver endoderm cells invade the STM and build liver buds *via* interactions with ECs. In addition, single-cell RNA sequence analysis of the organoids showed that the patterns of gene expression in ECs and STM within the organoids were more similar to those observed in human fetal liver than the patterns seen with the pre-mixed cells (Camp et al., 2017). This suggests that signaling within the organoids may contribute to bi-directional interaction of individual cells to approximate the human fetal liver. It is thus likely that mixing hepatic endodermal cells with cells in the liver-specific mesoderm lineage, including STM and ECs, to induce organogenesis during development may be an effective means of promoting organoid construction to a maturity similar to that of the adult liver.

An alternative approach to mixing cells from distinctive differentiation systems is the fusion of organoid, which reportedly promotes organogenesis during development (Kanton and Pasca, 2022). This differentiation system is based on the concept that joining several different tissues promotes local intercellular communication at their junction (Niessen et al., 2011). In a study focusing on the fact that liver development occurs from the border region between the anterior and posterior foregut, anterior and posterior foregut organoids were used to induce *PROX1-*expressing cells, the primordium of both liver and pancreas, from the junction region (Koike et al., 2019; Koike et al., 2021). This boundary region gradually buds into a hepato-biliary-pancreas organoid (HBPO) connected by bile duct structures. HBPOs can be applied to evaluate continuous organogenesis within organs, which has been difficult with single-tissue organoids. Further understanding of the complex mechanisms contributing to organogenesis during human organoid construction is expected to shed light on aspects of human developmental biology that are otherwise challenging to analyze.

### 2.2 Disease modeling

Liver diseases such as NASH and AFLD are known to progress through the coordinated actions of multiple cell types playing different roles. Although these pathological conditions can not be reproduced in pathological models composed of 2D cultures of single cells, the use of human PSC-derived organoids has made it possible to reproduce them *in vitro*. In NASH, for example, multiple cells, including hepatocytes that accumulate fat, Kupffer cells that induce inflammation, and hepatic stellate cells that are the source of fibrosis play central roles in the pathogenesis. In a study in which a multicellular liver organoid system composed mainly of hepatocytes, Kupffer cells, and hepatic stellate cells was constructed from PSCs in a stepwise protocol, the primary pathophysiology of NASH (fat accumulation, inflammation, fibrosis) was reproduced in a single well upon administration of oleic acid, a type of free fatty acid (Ouchi et al., 2019). In another study, liver organoids created in a multicellular system in the absence of Matrigel by inducing differentiation of PSCs into each of four cell types, including ECs, were shown to recapitulate several NASH pathologies (Tsang et al., 2021). In similar fashion, modeling of AFLD, in which fatty liver develops due to heavy and continuous alcohol intake and progresses to inflammation and fibrosis, was successfully established in a system of liver organoids co-cultured with fetal liver mesenchymal cells and human ESC-derived hepatic organoids (Wang et al., 2019). Upon treatment with ethanol, this model reproduced the pathogenesis of AFLD, including fat accumulation, elevated enzymes involved in ethanol metabolism, increased inflammation and fibrosis. Moreover, it was shown that a hepatobiliary organoid in which a bile canalicular network connected hepatocyte organoids and bile duct organoids could be created from PSCs in a Matrix-free state using a stepwise protocol and that this model reproduces the pathogenesis of inflammatory drug-induced liver injury (DILI) (Ramli et al., 2020). Treating these organoids with troglitazone, a diabetes drug known to cause DILI, induced cell death within a week. It also increased ALT levels, which is indicative of the inflammatory state of hepatocytes, and disrupted or eliminated the biliary canaliculi network, thereby inhibiting intercellular communication between hepatocytes and bile duct cells. In both culture systems, the presence of multiple cell types and an environment for cell-to-cell communication led to the reproduction of the pathology. An *in vitro* infection model with hepatitis B virus (HBV) has also been created using a liver organoid constructed by co-culturing iPSC-derived endoderm with HUVECs and MSCs in a 3D microwell system (Nie et al., 2018). The iPSC-derived liver organoid infected in this assay released significant amounts of virus and strongly expressed infection-promoting factors, demonstrating the stability of this infection model. It is anticipated that this model will serve as an effective tool for HBV research able to encompass the patient-specific genetic background and to show higher susceptibility to HBV infection than iPSC-derived hepatic-like cells.

One of the advantages of using iPSCs is that the genetic background of the patient can be reflected in the organoids. For example, liver organoids generated using iPSCs from Wolman disease patients, who develop fatty liver and cirrhosis due to a genetic defect in the enzyme lysosomal acid lipase, showed predominantly higher levels of fatty liver and fibrosis than organoids constructed with iPSCs from healthy individuals (Ouchi et al., 2019). This indicates that the genetic background of Wolman disease is reflected in the organoids. In addition, in a study using a human iPSC-derived liver organoid panel from 24 individuals with/without risk SNPs for NAFLD identified through a genome-wide association study (GWAS), GCKR (rs780094 C>T) was shown to be the SNP most strongly correlated with fat accumulation (Kimura et al., 2022). Interestingly, GCKR(TT) was associated with an increased risk of NAFLD in the presence of insulin resistance but a decreased risk in the absence of insulin resistance.

The combination of iPSC-based organoid systems and genome editing technology has revealed the pathogenesis of causative mutations in human genetic diseases. For instance, iPSCs from patients with deoxyguanosine kinase gene (*DGUOK*) mutations, a major cause of mitochondrial DNA depletion syndrome, were used to create liver organoids, a model that includes both hepatocyte characteristics and the patient’s genetic background (Guo et al., 2021). Compared to liver organoids in which the mutation was corrected through CRISPR-Cas9 gene editing, the *DGUOK* mutant was more susceptible to ferroptosis in response to iron overload. In another study using iPSC-derived liver organoids containing hepatocytes and cholangiocytes, CRISPR-Cas9 gene editing was used to evaluate the impact of NOTCH ligand *JAG1* mutation on development of Alagille syndrome, a genetic disorder where NOTCH signaling pathway mutations impair bile duct formation (Guan et al., 2017). With continued advancements in gene editing, such as with CRISPR-Cas9 (Hockemeyer and Jaenisch, 2016), it is anticipated that additional disease-reproducing liver organoids will be reported in the future. Such disease models are expected to enable elucidation of a disease’s pathological mechanism as well as individualized and stratified screening of drugs, leading to the development of new therapeutic agents targeting specific diseases in individual patients.

### 2.3 Drug discovery

Hepatocytes are the primary targets of drugs affecting metabolic function. Organoids constructed of hepatocytes are a valuable model for evaluating drug safety and efficacy because they ensure metabolic drug activity, resemble primary hepatocytes more than secondary culture systems, and are more sensitive to toxicity (Mun et al., 2019; Shinozawa et al., 2021). In hepatocyte organoids treated with oleic and palmitic acids and accumulated triglycerides, the addition of L-carnitine, a known anti-fatty liver drug, and metformin, an antidiabetic drug, has been shown to improve the morphology of oleic and palmitic acid-treated organoids and to substantially reduce triglycerides (Mun et al., 2019). In addition, anti-steatosis drug screening based on high-content analysis of Nile red staining showed that several drugs reduced triglycerides, indicating that hepatocyte organoids are helpful for drug validation. Furthermore, a high-speed live imaging platform for DILI has been established using hepatocyte organoids with polarity similar to human hepatocytes (Shinozawa et al., 2021). This 384-well-based system enables cell visualization and evaluation of pharmacokinetics and survival in real-time. With this system more than 450 new and existing drugs were administered to polar human liver organoids, and drugs that cause biliary congestion were identified. Liver organoids have also been generated from multiple iPSC lines with/without *CYP2C9* mutations, and the effect of the drug Bosentan, which causes cholestasis, was verified. Notably, it was found that genetic variants may alter the action of Bosentan and patient-specific responses. In another study, cholangiocyte organoids were used to investigate the efficacy of drugs targeting cystic fibrosis-associated liver diseases (Sampaziotis et al., 2015). Using patient-derived iPSC organoids, the effects of verapamil and octreotide on polycystic and cystic fibrosis liver disease were reproduced, suggesting the utility of patient-derived organoids for drug screening.

The finding summarizes above suggest that if a simple and stable organoid induction technique can be developed, the constructed iPSC-derived organoids could serve as high-throughput drug evaluation systems able to provide novel therapeutic agents and diagnostic markers as well as personalized treatments.

### 2.4 Regenerative medicine

Although the liver is a regenerative organ, living donor liver transplantation is the only treatment for severe liver damage. It is estimated that more than 30,000 patients go on the liver transplant waiting list each year in the United States (Kwong et al., 2021), and the shortage of organ donors for transplantation is a serious problem. Because liver organoids have been reported to have better successful rate of engraftment than 2D hepatocytes (Takebe et al., 2014; Stevens et al., 2017), the ability to transplant healthy patient-specific liver organoids could potentially restore organ function. In mice, for example, 3D liver organoids pre-impregnated with vascular-like structures by mixing in self-aggregating vascular ECs are able to connect to host blood vessels within 2 days after transplantation and improve survival after acute liver injury (Takebe et al., 2013). In this mouse model, liver organoids provided an excellent engraftment rate and growth promotion as well as long-term survival. Similar results were obtained with liver organoids containing pre-patterned human primary hepatocytes, ECs, and stromal cells in degradable hydrogel (Stevens et al., 2017). Because replacement of liver function requires more than 100,000,000 cells, culture systems are beginning to be established to provide sufficient cell numbers through progenitor cell proliferation methods (Koike et al., 2017; Zhang et al., 2018) and miniaturization of liver organoids (Takebe et al., 2017a). In addition, a differentiation induction system using chemically defined medium has been established, and an organoid culture method with excellent reproducibility and safety has been developed (Sekine et al., 2020).

One option envisioned for regenerative medicine using iPSCs is autologous transplantation, which is less prone to immune rejection. On the other hand, regenerative medicine through xenotransplantation using other people’s cells inevitably involves the risk of immune rejection if the HLA types the donor and recipient cells differ. Currently, iPSCs that are HLA homozygous combinations less likely to cause rejection (Umekage et al., 2019) are being stored in the Center for iPS Cell Research and Application at Kyoto University, and the use of genome editing to create HLA pseudo-homozygotes and HLA-C-positive cells that are less susceptible to attack from killer T cells and NK cells (Xu et al., 2019) has been completed, expecting this approach available for numerous people worldwide. The safety of human iPSC-derived liver organoids for transplantation to infant patients has been evaluated using a pig model (Tsuchida et al., 2020), and studies evaluating human transplantation of iPSC-derived organoids, including the liver, are progressing.

## 3 Discussion

Organoid technology using PSCs, especially iPSCs, is expected to play a key role in a variety of fields, including embryology, pathological modeling, drug screening, and regenerative medicine. However, several issues remain to be addressed. The first is the reproducibility of organoid formation. Although biochemical differentiation protocols have become more efficient, more robust protocols are needed to reduce output variation when starting from different cell lines. In that regard, the stepwise induction protocol for human liver organoids generated from 11 different donor iPSCs (Ouchi et al., 2019) is a highly reproducible protocol, the utility of which was confirmed in later differentiation experiments with iPSCs from 10 to 24 different donors (Shinozawa et al., 2021; Kimura et al., 2022). The second issue is the immaturity of organoids. So far, no PSC-derived liver organoids have reached adult-level organ maturity *ex vivo*, and significant improvement of the technique is still required. For instance, when a microfluidics system called an “organ on a chip,” which produces anaerobic exchange substance and shear stress, was used to reproduce bile and vascular flow, the complexity of the structure and metabolic functions of a human liver-related, cell line-derived organoid were improved (Rennert et al., 2015). As for organ-on-a-chip, it has also been shown to be useful in faithfully reproducing human pathology in multiple organs (Hiratsuka et al., 2022; Ingber, 2022). In another example, using bioprinting to change the shape of a 3D-printed gelatin scaffold improved the function of a human hepatocyte line (Lewis et al., 2018). In human iPSC-derived intestinal organoids, circadian rhythms were observed upon differentiation and maturation and indeed, it has been suggested that *in vitro* introduction of circadian feeding and fasting cycles in pancreatic islets induces the cyclic synthesis and maturation of energy metabolism and insulin secretory effectors (Alvarez-Dominguez et al., 2020). In addition, two adult hepatocyte-specific transcription factors, NFIX and NFIA, were identified through single RNA sequencing analysis comparing adult hepatocytes, human fetal hepatocytes, and human iPSC-derived hepatocyte-like cells, and their overexpression during the induction process from human iPSCs to hepatocyte-like cells was found to promote the cells’ maturation (Wesley et al., 2022). These suggest that factors related to cell maturation may be strong factors promoting maturity on the PSC-derived organoid system. The third issue is the difference in structural complexity between organoids and liver tissue. PSC-derived organoids are often constructed with many more cell types than 2D cultures or primary cell-derived organoids, but they have yet to fully reproduce the complexity of the cellular society within a living organ. For example, no liver organoid that contains nerves, lymphocytes, and other immune cells yet exists. Thus, more advanced organoids will likely need to be developed in the future. In addition, along with the increasing complexity of single organoid structures, there are growing expectations for the need to model their relationships with surrounding organs (Takebe et al., 2017b). For instance, iPSC-derived liver and islet organoids can be sustained for 30 days in a co-cultured multi-organoid model using an “organ on a chip” microfluidic system (Tao et al., 2022). It has been suggested that this model could be applied to evaluate the functional relationships between the liver and islets in response to external hyperglycemic stimuli and drugs. It is anticipated that further development of “organ on a chip” technology will expand the evaluation system for human interorgan coordination, which has not been achieved in animal studies. The fourth issue is the use of animal-derived matrices for organoid creation. Embedding organoids in Matrigel or other animal-derived extracellular matrix components used as scaffolds induces cell self-assembly and maturation. However, these extracellular matrix materials are poorly characterized and, therefore, unsuitable for clinical applications such as regenerative medicine. They also complicate genetic manipulation and organoid passaging. The recent development of Matrigel-free organoids and extracellular substrates, such as inverted colloidal crystals with polyethylene glycol (Ng et al., 2018) and synthetic hydrogels based on polyisocyanopeptides (Ye et al., 2020), is expected to enable development of suitable technological methods for construction of liver organoids for regenerative medicine (Kozlowski et al., 2021).

In conclusion, this review has outlined the latest research on functional liver organoids derived from PSCs. It was not mentioned in this review but organoids that recapitulate many organs and diseases have already been created around the world (Kim et al., 2020). Along with the resolution of the issue mentioned in the previous paragraph, PSC-derived organoid research will be widely used in many fields of study and the techniques and findings discovered in these organoid systems will 1 day be applied to many human patients.

## References

[B1] Alvarez-Dominguez J. R. Donaghey J. Rasouli N. Kenty J. H. R. Helman A. Charlton J. (2020). Circadian entrainment triggers maturation of human *in vitro* islets. Cell Stem Cell 26 (1), 108–122.e10. 10.1016/j.stem.2019.11.011 31839570

[B2] Ardalani H. Sengupta S. Harms V. Vickerman V. Thomson J. A. Murphy W. L. (2019). 3-D culture and endothelial cells improve maturity of human pluripotent stem cell-derived hepatocytes. Acta Biomater. 95, 371–381. 10.1016/j.actbio.2019.07.047 31362140

[B3] Armingol E. Officer A. Harismendy O. Lewis N. E. (2021). Deciphering cell-cell interactions and communication from gene expression. Nat. Rev. Genet. 22 (2), 71–88. 10.1038/s41576-020-00292-x 33168968 PMC7649713

[B4] Asai A. Aihara E. Watson C. Mourya R. Mizuochi T. Shivakumar P. (2017). Paracrine signals regulate human liver organoid maturation from induced pluripotent stem cells. Development 144 (6), 1056–1064. 10.1242/dev.142794 28275009 PMC5358109

[B5] Camp J. G. Sekine K. Gerber T. Loeffler-Wirth H. Binder H. Gac M. (2017). Multilineage communication regulates human liver bud development from pluripotency. Nature 546 (7659), 533–538. 10.1038/nature22796 28614297

[B6] El Hokayem J. Cukier H. N. Dykxhoorn D. M. (2016). Blood derived induced pluripotent stem cells (iPSCs): Benefits, challenges and the road ahead. J. Alzheimers Dis. Park. 6 (5), 275. 10.4172/2161-0460.1000275 PMC5118044 27882265

[B7] Guan Y. Xu D. Garfin P. M. Ehmer U. Hurwitz M. Enns G. (2017). Human hepatic organoids for the analysis of human genetic diseases. JCI Insight 2 (17), e94954. 10.1172/jci.insight.94954 28878125 PMC5621886

[B8] Guo J. Duan L. He X. Li S. Wu Y. Xiang G. (2021). A combined model of human iPSC-derived liver organoids and hepatocytes reveals ferroptosis in DGUOK mutant mtDNA depletion syndrome. Adv. Sci. (Weinh) 8 (10), 2004680. 10.1002/advs.202004680 34026460 PMC8132052

[B9] Han L. Chaturvedi P. Kishimoto K. Koike H. Nasr T. Iwasawa K. (2020). Single cell transcriptomics identifies a signaling network coordinating endoderm and mesoderm diversification during foregut organogenesis. Nat. Commun. 11 (1), 4158. 10.1038/s41467-020-17968-x 32855417 PMC7453027

[B10] Hiratsuka K. Miyoshi T. Kroll K. T. Gupta N. R. Valerius M. T. Ferrante T. (2022). Organoid-on-a-chip model of human ARPKD reveals mechanosensing pathomechanisms for drug discovery. Sci. Adv. 8 (38), eabq0866. 10.1126/sciadv.abq0866 36129975 PMC9491724

[B11] Hockemeyer D. Jaenisch R. (2016). Induced pluripotent stem cells meet genome editing. Cell Stem Cell 18 (5), 573–586. 10.1016/j.stem.2016.04.013 27152442 PMC4871596

[B12] Ingber D. E. (2022). Human organs-on-chips for disease modelling, drug development and personalized medicine. Nat. Rev. Genet. 23 (8), 467–491. 10.1038/s41576-022-00466-9 35338360 PMC8951665

[B13] Kanton S. Pasca S. P. (2022). Human assembloids. Development 149 (20), dev201120. 10.1242/dev.201120 36317797

[B14] Kim J. Koo B. K. Knoblich J. A. (2020). Human organoids: Model systems for human biology and medicine. Nat. Rev. Mol. Cell Biol. 21 (10), 571–584. 10.1038/s41580-020-0259-3 32636524 PMC7339799

[B15] Kimura M. Iguchi T. Iwasawa K. Dunn A. Thompson W. L. Yoneyama Y. (2022). En masse organoid phenotyping informs metabolic-associated genetic susceptibility to NASH. Cell 185 (22), 4216–4232.e16. 10.1016/j.cell.2022.09.031 36240780 PMC9617783

[B16] Koike H. Iwasawa K. Ouchi R. Maezawa M. Giesbrecht K. Saiki N. (2019). Modelling human hepato-biliary-pancreatic organogenesis from the foregut-midgut boundary. Nature 574 (7776), 112–116. 10.1038/s41586-019-1598-0 31554966 PMC7643931

[B17] Koike H. Iwasawa K. Ouchi R. Maezawa M. Kimura M. Kodaka A. (2021). Engineering human hepato-biliary-pancreatic organoids from pluripotent stem cells. Nat. Protoc. 16 (2), 919–936. 10.1038/s41596-020-00441-w 33432231 PMC8212777

[B18] Koike H. Zhang R. R. Ueno Y. Sekine K. Zheng Y. W. Takebe T. (2017). Nutritional modulation of mouse and human liver bud growth through a branched-chain amino acid metabolism. Development 144 (6), 1018–1024. 10.1242/dev.143032 28219950

[B19] Koui Y. Kido T. Ito T. Oyama H. Chen S. W. Katou Y. (2017). An *in vitro* human liver model by iPSC-derived parenchymal and non-parenchymal cells. Stem Cell Rep. 9 (2), 490–498. 10.1016/j.stemcr.2017.06.010 PMC5549957 28757162

[B20] Kozlowski M. T. Crook C. J. Ku H. T. (2021). Towards organoid culture without Matrigel. Commun. Biol. 4 (1), 1387. 10.1038/s42003-021-02910-8 34893703 PMC8664924

[B21] Kwong A. J. Kim W. R. Lake J. R. Smith J. M. Schladt D. P. Skeans M. A. (2021). OPTN/SRTR 2019 annual data report: Liver. Am. J. Transpl. 21, 208–315. 10.1111/ajt.16494 33595192

[B22] Lewis P. L. Green R. M. Shah R. N. (2018). 3D-printed gelatin scaffolds of differing pore geometry modulate hepatocyte function and gene expression. Acta Biomater. 69, 63–70. 10.1016/j.actbio.2017.12.042 29317370 PMC5831494

[B23] Margagliotti S. Clotman F. Pierreux C. E. Beaudry J. B. Jacquemin P. Rousseau G. G. (2007). The Onecut transcription factors HNF-6/OC-1 and OC-2 regulate early liver expansion by controlling hepatoblast migration. Dev. Biol. 311 (2), 579–589. 10.1016/j.ydbio.2007.09.013 17936262

[B24] Miyoshi M. Kakinuma S. Kamiya A. Tsunoda T. Tsuchiya J. Sato A. (2019). LIM homeobox 2 promotes interaction between human iPS-derived hepatic progenitors and iPS-derived hepatic stellate-like cells. Sci. Rep. 9 (1), 2072. 10.1038/s41598-018-37430-9 30765795 PMC6376133

[B25] Morizane A. Kikuchi T. Hayashi T. Mizuma H. Takara S. Doi H. (2017). MHC matching improves engraftment of iPSC-derived neurons in non-human primates. Nat. Commun. 8 (1), 385. 10.1038/s41467-017-00926-5 28855509 PMC5577234

[B26] Mun S. J. Ryu J. S. Lee M. O. Son Y. S. Oh S. J. Cho H. S. (2019). Generation of expandable human pluripotent stem cell-derived hepatocyte-like liver organoids. J. Hepatol. 71 (5), 970–985. 10.1016/j.jhep.2019.06.030 31299272

[B27] Ng S. S. Saeb-Parsy K. Blackford S. J. I. Segal J. M. Serra M. P. Horcas-Lopez M. (2018). Human iPS derived progenitors bioengineered into liver organoids using an inverted colloidal crystal poly (ethylene glycol) scaffold. Biomaterials 182, 299–311. 10.1016/j.biomaterials.2018.07.043 30149262 PMC6131727

[B28] Nie Y. Z. Zheng Y. W. Miyakawa K. Murata S. Zhang R. R. Sekine K. (2018). Recapitulation of Hepatitis B virus-host interactions in liver organoids from human induced pluripotent stem cells. EBioMedicine 35, 114–123. 10.1016/j.ebiom.2018.08.014 30120080 PMC6156717

[B29] Niessen C. M. Leckband D. Yap A. S. (2011). Tissue organization by cadherin adhesion molecules: Dynamic molecular and cellular mechanisms of morphogenetic regulation. Physiol. Rev. 91 (2), 691–731. 10.1152/physrev.00004.2010 21527735 PMC3556819

[B30] Ouchi R. Togo S. Kimura M. Shinozawa T. Koido M. Koike H. (2019). Modeling steatohepatitis in humans with pluripotent stem cell-derived organoids. Cell Metab. 30 (2), 374–384.e6. 10.1016/j.cmet.2019.05.007 31155493 PMC6687537

[B31] Raggi C. M'Callum M. A. Pham Q. T. Gaub P. Selleri S. Baratang N. V. (2022). Leveraging interacting signaling pathways to robustly improve the quality and yield of human pluripotent stem cell-derived hepatoblasts and hepatocytes. Stem Cell Rep. 17 (3), 584–598. 10.1016/j.stemcr.2022.01.003 PMC9039749 35120625

[B32] Ramli M. N. B. Lim Y. S. Koe C. T. Demircioglu D. Tng W. Gonzales K. A. U. (2020). Human pluripotent stem cell-derived organoids as models of liver disease. Gastroenterology 159 (4), 1471–1486.e12. 10.1053/j.gastro.2020.06.010 32553762

[B33] Reinke H. Asher G. (2019). Crosstalk between metabolism and circadian clocks. Nat. Rev. Mol. Cell Biol. 20 (4), 227–241. 10.1038/s41580-018-0096-9 30635659

[B34] Rennert K. Steinborn S. Groger M. Ungerbock B. Jank A. M. Ehgartner J. (2015). A microfluidically perfused three dimensional human liver model. Biomaterials 71, 119–131. 10.1016/j.biomaterials.2015.08.043 26322723

[B35] Sampaziotis F. de Brito M. C. Madrigal P. Bertero A. Saeb-Parsy K. Soares F. A. C. (2015). Cholangiocytes derived from human induced pluripotent stem cells for disease modeling and drug validation. Nat. Biotechnol. 33 (8), 845–852. 10.1038/nbt.3275 26167629 PMC4768345

[B36] Sekine K. Ogawa S. Tsuzuki S. Kobayashi T. Ikeda K. Nakanishi N. (2020). Generation of human induced pluripotent stem cell-derived liver buds with chemically defined and animal origin-free media. Sci. Rep. 10 (1), 17937. 10.1038/s41598-020-73908-1 33087763 PMC7578079

[B37] Shinozawa T. Kimura M. Cai Y. Saiki N. Yoneyama Y. Ouchi R. (2021). High-fidelity drug-induced liver injury screen using human pluripotent stem cell-derived organoids. Gastroenterology 160 (3), 831–846.e10. 10.1053/j.gastro.2020.10.002 33039464 PMC7878295

[B38] Sosa-Pineda B. Wigle J. T. Oliver G. (2000). Hepatocyte migration during liver development requires Prox1. Nat. Genet. 25 (3), 254–255. 10.1038/76996 10888866

[B39] Stevens K. R. Scull M. A. Ramanan V. Fortin C. L. Chaturvedi R. R. Knouse K. A. (2017). *In situ* expansion of engineered human liver tissue in a mouse model of chronic liver disease. Sci. Transl. Med. 9 (399), eaah5505. 10.1126/scitranslmed.aah5505 28724577 PMC5896001

[B40] Takahashi K. Tanabe K. Ohnuki M. Narita M. Ichisaka T. Tomoda K. (2007). Induction of pluripotent stem cells from adult human fibroblasts by defined factors. Cell 131 (5), 861–872. 10.1016/j.cell.2007.11.019 18035408

[B41] Takebe T. Sekine K. Enomura M. Koike H. Kimura M. Ogaeri T. (2013). Vascularized and functional human liver from an iPSC-derived organ bud transplant. Nature 499 (7459), 481–484. 10.1038/nature12271 23823721

[B42] Takebe T. Sekine K. Kimura M. Yoshizawa E. Ayano S. Koido M. (2017a). Massive and reproducible production of liver buds entirely from human pluripotent stem cells. Cell Rep. 21 (10), 2661–2670. 10.1016/j.celrep.2017.11.005 29212014

[B43] Takebe T. Zhang B. Radisic M. (2017b). Synergistic engineering: Organoids meet organs-on-a-chip. Cell Stem Cell 21 (3), 297–300. 10.1016/j.stem.2017.08.016 28886364

[B44] Takebe T. Zhang R. R. Koike H. Kimura M. Yoshizawa E. Enomura M. (2014). Generation of a vascularized and functional human liver from an iPSC-derived organ bud transplant. Nat. Protoc. 9 (2), 396–409. 10.1038/nprot.2014.020 24457331

[B45] Tao T. Deng P. Wang Y. Zhang X. Guo Y. Chen W. (2022). Microengineered multi-organoid system from hiPSCs to recapitulate human liver-islet Axis in normal and type 2 diabetes. Adv. Sci. (Weinh) 9 (5), e2103495. 10.1002/advs.202103495 34951149 PMC8844474

[B46] Thomson J. A. Itskovitz-Eldor J. Shapiro S. S. Waknitz M. A. Swiergiel J. J. Marshall V. S. (1998). Embryonic stem cell lines derived from human blastocysts. Science 282 (5391), 1145–1147. 10.1126/science.282.5391.1145 9804556

[B47] Touboul T. Hannan N. R. Corbineau S. Martinez A. Martinet C. Branchereau S. (2010). Generation of functional hepatocytes from human embryonic stem cells under chemically defined conditions that recapitulate liver development. Hepatology 51 (5), 1754–1765. 10.1002/hep.23506 20301097

[B48] Tsang H. Y. Yi Lo P. H. Ho Lee K. K. (2021). “Generation of liver organoids from human induced pluripotent stem cells as liver fibrosis and steatosis models,”. bioRxiv. 2021.2006.2029.450347.

[B49] Tsuchida T. Murata S. Hasegawa S. Mikami S. Enosawa S. Hsu H. C. (2020). Investigation of clinical safety of human iPS cell-derived liver organoid transplantation to infantile patients in porcine model. Cell Transpl. 29, 963689720964384. 10.1177/0963689720964384 PMC7784600 33103476

[B50] Umekage M. Sato Y. Takasu N. (2019). Overview: An iPS cell stock at CiRA. Inflamm. Regen. 39, 17. 10.1186/s41232-019-0106-0 31497180 PMC6717959

[B51] Wang S. Wang X. Tan Z. Su Y. Liu J. Chang M. (2019). Human ESC-derived expandable hepatic organoids enable therapeutic liver repopulation and pathophysiological modeling of alcoholic liver injury. Cell Res. 29 (12), 1009–1026. 10.1038/s41422-019-0242-8 31628434 PMC6951343

[B52] Wesley B. T. Ross A. D. B. Muraro D. Miao Z. Saxton S. Tomaz R. A. (2022). Single-cell atlas of human liver development reveals pathways directing hepatic cell fates. Nat. Cell Biol. 24 (10), 1487–1498. 10.1038/s41556-022-00989-7 36109670 PMC7617064

[B53] Xu H. Wang B. Ono M. Kagita A. Fujii K. Sasakawa N. (2019). Targeted disruption of HLA genes via CRISPR-cas9 generates iPSCs with enhanced immune compatibility. Cell Stem Cell 24 (4), 566–578.e7. 10.1016/j.stem.2019.02.005 30853558

[B54] Ye S. Boeter J. W. B. Mihajlovic M. van Steenbeek F. G. van Wolferen M. E. Oosterhoff L. A. (2020). A chemically defined hydrogel for human liver organoid culture. Adv. Funct. Mater 30 (48), 2000893. 10.1002/adfm.202000893 34658689 PMC7611838

[B55] Zaret K. S. (2002). Regulatory phases of early liver development: Paradigms of organogenesis. Nat. Rev. Genet. 3 (7), 499–512. 10.1038/nrg837 12094228

[B56] Zhang R. R. Koido M. Tadokoro T. Ouchi R. Matsuno T. Ueno Y. (2018). Human iPSC-derived posterior gut progenitors are expandable and capable of forming gut and liver organoids. Stem Cell Rep. 10 (3), 780–793. 10.1016/j.stemcr.2018.01.006 PMC5919071 29429958

[B57] Zorn A. M. (2008). “Liver development,” in StemBook (Cambridge (MA): Workman Publishing).

